# Atazanavir/Ritonavir-Associated Erythema Multiforme in an HIV-1-Positive Patient

**DOI:** 10.7759/cureus.115007

**Published:** 2026-08-22

**Authors:** Ranjan K Singh

**Affiliations:** 1 Medicine, Jai Prakash Narayan Hospital, Gaya, IND

**Keywords:** atazanavir, erythema multiforme (em), hiv-1, protease inhibitor, stevens-johnson syndrome (sjs)

## Abstract

We report a case of erythema multiforme (EM) associated with atazanavir/ritonavir in a patient with HIV-1 infection. The patient was receiving tenofovir, lamivudine, and dolutegravir; however, owing to a supply chain issue, dolutegravir was substituted with atazanavir/ritonavir. The patient subsequently developed pruritic erythematous papules and plaques with characteristic target lesions over the face and a hemorrhagic crust on the lower lip, without epidermal detachment or systemic symptoms. The Naranjo probability score indicated a probable adverse drug reaction. This case highlights the importance of recognizing EM as a potential adverse reaction to atazanavir and of early drug withdrawal to prevent progression to more severe cutaneous adverse reactions, including Stevens-Johnson syndrome.

## Introduction

Erythema multiforme (EM) is an acute immune-mediated disorder characterized by distinctive target lesions, typically distributed symmetrically over the extremities. Mucosal involvement may occur, particularly in EM major. Infections, especially herpes simplex virus (HSV), are the most frequently recognized triggers, while Mycoplasma pneumoniae has also been implicated. The estimated prevalence of EM ranges from 0.01% to 1% [1]. Drug-induced EM is less prevalent, whereas cutaneous rashes associated with atazanavir have been reported in approximately 1%-6% of patients [2].

Atazanavir is an HIV-1 protease inhibitor used in combination with other antiretroviral agents for the treatment of HIV-1 infection. It is generally well tolerated; characteristic adverse effects include indirect hyperbilirubinemia, jaundice, and cutaneous reactions. Cutaneous adverse reactions usually occur during the early period after treatment initiation and may range from mild maculopapular eruptions to severe cutaneous adverse reactions.

The prescribing information for atazanavir (REYATAZ®) lists serious cutaneous reactions, including EM, Stevens-Johnson syndrome (SJS), and toxic epidermal necrolysis (TEN) [3]. However, clinically documented cases of EM associated with atazanavir or atazanavir/ritonavir appear to be uncommon compared with reports of nonspecific or maculopapular cutaneous eruptions [2,4].

The Naranjo Adverse Drug Reaction Probability Scale is a commonly used tool that evaluates factors including temporal association, alternative causes, de-challenge, re-challenge, dose-response relationships, and previous reports of the reaction [5].

We report a case of EM developing shortly after initiation of atazanavir/ritonavir in an HIV-1-positive patient. The case highlights the characteristic clinical morphology, temporal relationship with treatment initiation, resolution following drug withdrawal, and the attribution of the target eruptions to the medication.

## Case presentation

**Figure 1:**
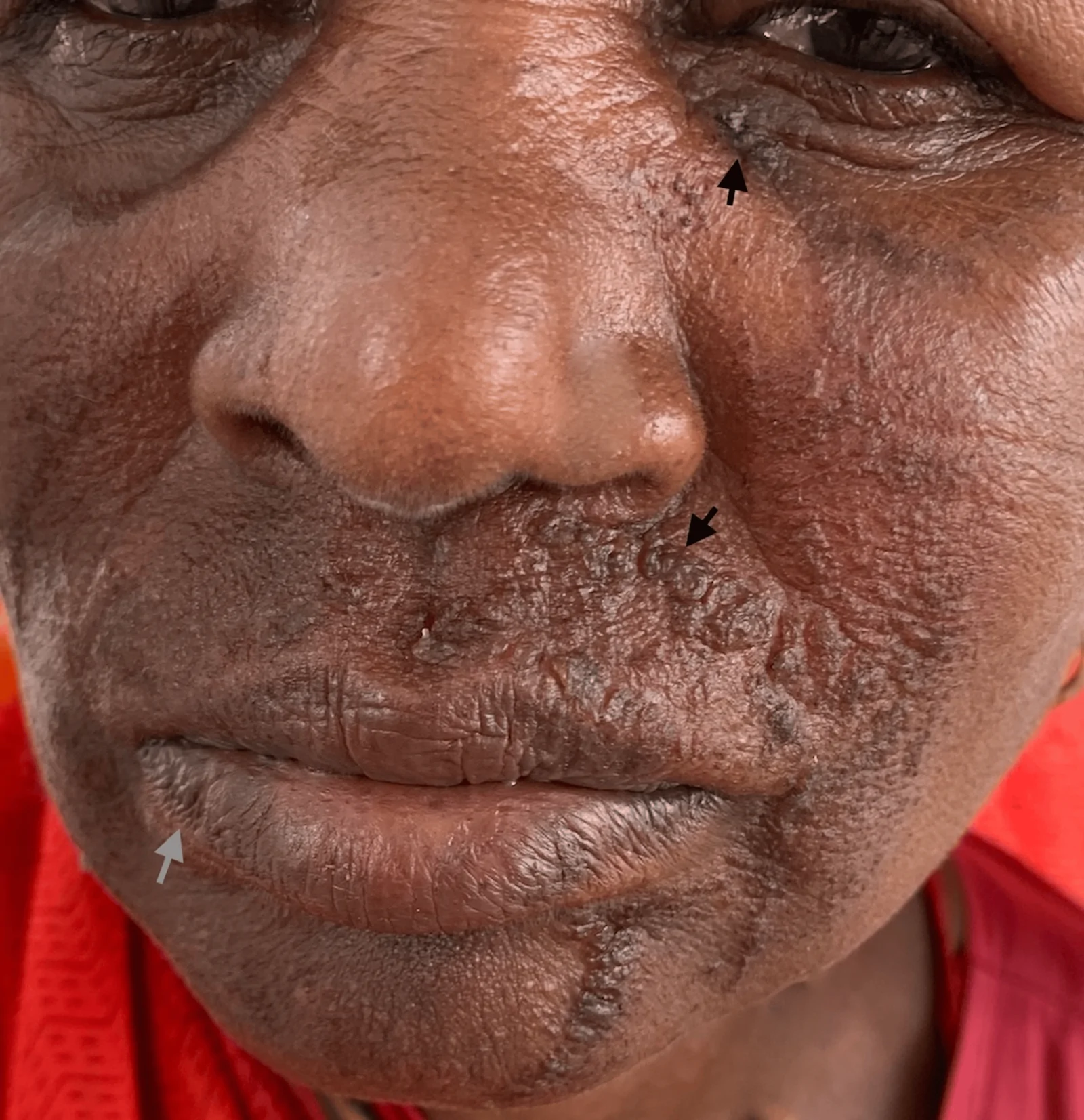
Target lesions of erythema multiforme involving face, including lesions in the left infraorbital region and left nasolabial fold (black arrows); a hemorrhagic crust on the lower lip (white arrow)

A 40-year-old woman with HIV-1 infection had been clinically stable on an antiretroviral regimen containing dolutegravir for five years, with sustained virologic suppression (Table 1). There was no evidence of hepatitis B or C co-infection. Because of an unforeseen medication supply issue, dolutegravir was replaced with atazanavir/ritonavir (300 mg/100 mg) once daily. On the third day after initiation of atazanavir/ritonavir, she developed pruritic erythematous papules and plaques, predominantly involving the face, with sparse lesions over the extremities (Figure 1).

On examination, characteristic target lesions were identified on the left nasolabial fold and inferior to the left lower eyelid. A hemorrhagic crust was also noted over the lower lip. There was no history of fever, preceding febrile illness, herpes labialis, or other clinically apparent infection. No ocular or genital involvement was noted, and there was no evidence of widespread epidermal detachment.

Laboratory investigations are summarized in Table 1. The findings were notable for mild eosinophilia, with no other significant abnormalities. 

**Table 1:** Laboratory findings.

Parameter	Patient value	Reference range
White blood cell (WBC) count	4,500/mm³	4,000-11,000/mm³
Neutrophils	56%	40%-75%
Eosinophils	9%	2%-6%
Platelet count	154 x 1,000/cmm	150-450 x 1,000/cmm
Serum bilirubin	0.70 mg/dL	0.2-1.2 mg/dL
Serum creatinine	0.8 mg/dL	0.7-1.4 mg/dL
Serum alanine aminotransferase (ALT)	35 U/L	7-50 U/L
HIV-1 RNA (viral load)	<20 copies/mL	<20 copies/mL

Based on the characteristic target lesions, clinical morphology, and temporal association with initiation of atazanavir/ritonavir, a clinical diagnosis of EM was made. The hemorrhagic crust over the lower lip raised the possibility of limited mucocutaneous involvement; however, there was no evidence of extensive mucosal disease. The absence of epidermal detachment and other features of severe mucocutaneous involvement argued against SJS or TEN.

Atazanavir/ritonavir was immediately discontinued, and an oral antihistamine was prescribed for symptomatic relief of pruritus. The patient was switched back to her previous antiretroviral regimen of tenofovir, lamivudine, and dolutegravir. The cutaneous lesions progressively resolved over two weeks, with no recurrence thereafter. The clinical timeline is presented in Table 2.

**Table 2:** Clinical timeline of probable atazanavir/ritonavir-associated erythema multiforme (EM).

Timeline	Medication/intervention	Adverse events	Outcome
Day 0	Atazanavir/ritonavir 300 mg/100 mg once daily initiated, replacing dolutegravir	-	-
Day 3	Atazanavir/ritonavir discontinued; oral antihistamine and oral hydration initiated	Pruritic erythematous papules and plaques with target lesions on the face and a hemorrhagic crust on the lower lip	EM; no widespread epidermal detachment or ocular/genital involvement
Following days	Previous antiretroviral regimen of tenofovir, lamivudine, and dolutegravir resumed	Cutaneous lesions progressively regressed	Clinical improvement
Two weeks	-	Complete clinical resolution of lesions	No residual scarring or pigmentation
Two-year follow-up	Continued previous antiretroviral regimen	No recurrence of cutaneous lesions	No recurrence or delayed cutaneous/systemic complications

The Naranjo algorithm yielded a score of 7, indicating a probable adverse drug reaction. The score was supported by the temporal relationship with drug initiation (+2), improvement after drug withdrawal (+1), absence of an identified alternative clinical trigger (+2), previous reports of cutaneous reactions (+1), and objective clinical evidence (+1). No re-challenge, placebo challenge, drug-level measurement, dose-response assessment, or previous similar reaction was documented (0 for each). However, because atazanavir and ritonavir were initiated concomitantly, the Naranjo algorithm cannot determine which individual drug was responsible.

## Discussion

EM is characterized clinically by the development of typical target lesions, often with an acral and symmetric distribution. The condition is most frequently associated with infections, particularly HSV, whereas drug-induced EM represents a relatively uncommon cause. Demouche et al. analysed reports of EM in the French pharmacovigilance database and found that only a small proportion were considered probable drug-induced cases, emphasizing the relatively uncommon role of medications as triggers [6].

Atazanavir has been associated with maculopapular eruptions; however, the prescribing information also notes the potential for severe cutaneous reactions, including EM, SJS, and TEN [2-4]. Despite this, clinical case reports that connect EM to atazanavir/ritonavir appear to be infrequent. Published case reports of atazanavir-associated severe cutaneous adverse reactions, along with relevant post-marketing safety information from the prescribing information, are summarized in Table 3.

Several features in the present case support an association between the eruption and the initiation of atazanavir/ritonavir. First, the patient had tolerated her previous antiretroviral regimen for five years without a similar reaction. Second, the eruption developed shortly after the introduction of atazanavir/ritonavir. Third, the lesions had characteristic target morphology. Fourth, the eruption progressively resolved after withdrawal of the newly introduced medication. Finally, the patient was able to resume her previous antiretroviral regimen without recurrence. Drug-induced EM typically arises from a Type IV T-cell-mediated hypersensitivity reaction, requiring a 7- to 21-day latency period for initial sensitization. A three-day onset indicates a possible anamnestic memory T-cell response stemming from prior exposure or sensitization. 

**Table 3:** Cutaneous adverse reactions associated with atazanavir or atazanavir/efavirenz. HIV, human immunodeficiency virus; EM, erythema multiforme; SJS, Stevens-Johnson syndrome; TEN, toxic epidermal necrolysis

Author/Source	Patient details	Latency	Clinical presentation	Reported diagnosis/severity	Management	Outcome
Walkty et al. (2009) [2]	Three HIV-positive patients	8-11 days	Pruritic maculopapular rash	Severe cutaneous rash; not specifically diagnosed as EM/SJS/TEN	Atazanavir discontinued; supportive treatment varied	Rash resolved after discontinuation
Irungu (2019) [7]	HIV-positive patient	A few days*	Vesiculobullous/targetoid lesions	Severe cutaneous reaction	Withdrawal of suspected antiretroviral therapy and supportive care	Recovery
REYATAZ prescribing information (2024) [3]	Post-marketing reports	Not individually reported	Not individually characterized	EM, SJS, and toxic skin eruptions have been reported	Discontinuation recommended if severe rash develops	Individual outcomes not reported
Present case	40-year-old woman with HIV-1 infection	3 days	Erythematous papules and plaques with target lesions; hemorrhagic crust of the lower lip	EM	Withdrawal of atazanavir/ritonavir and supportive treatment	Complete remission within two weeks

The hemorrhagic crust on the lower lip warrants particular attention. Although this finding may suggest limited mucosal involvement, there was no evidence of extensive mucosal disease, ocular involvement, genital involvement, or epidermal detachment. The overall clinical picture was therefore more consistent with EM than with SJS/TEN.

EM can clinically mimic various other skin conditions, which underscores the necessity for precise diagnosis. Differential diagnoses include TEN resembling lupus-Lyell syndrome, bullous drug reactions, and infectious rashes such as hand-foot-and-mouth disease. Additionally, Rowell syndrome, a rare skin manifestation linked to lupus erythematosus, may also present with EM-like targetoid lesions and should be taken into account in the relevant clinical context [8].

This case has a few limitations. Atazanavir and ritonavir were initiated concomitantly; therefore, the individual drug responsible cannot be determined. Infectious triggers were not investigated by laboratory testing and cannot be definitively excluded, although no clinically apparent infectious trigger was identified. Specific investigations for HSV, including PCR or serology, and Mycoplasma pneumoniae were not performed during the acute presentation. No re-challenge was performed because of the potential risk of recurrence or a more severe reaction. 

## Conclusions

This case highlights the significance of identifying EM as a probable cutaneous adverse reaction linked to atazanavir/ritonavir. While infections, especially those caused by HSV and M. pneumoniae, are more frequently recognized as triggers for EM, the close timing between the start of atazanavir/ritonavir and the appearance of the rash, along with the complete resolution after discontinuation of the medication, indicates a probable drug-related connection.
